# An Ultra-Wide Band Antenna System for Pulsed Sources Direction Finding †

**DOI:** 10.3390/s20174695

**Published:** 2020-08-20

**Authors:** Razvan D. Tamas, Stefania Bucuci

**Affiliations:** Department of Electronics and Telecommunications, Constanta Maritime University, 900663 Constanta, Romania; tamas@ieee.org

**Keywords:** ultra-wide band antennas, energy-based antenna descriptors, pulsed sources, direction finding

## Abstract

Electric discharges in high-voltage power distribution systems can be localized through their electromagnetic signature in the radio-frequency range. Since discharges produce series of short pulses, the corresponding spectrum usually covers wide frequency ranges, typically up to 1 GHz. In this paper, we propose an ultra-wide band (UWB) antenna system and a direction-finding (DF) approach based on using energy-based descriptors, instead of classical frequency-domain parameters. As an antenna system, we propose a dual-padlock configuration with a suitable pulse-matched response, featuring two unbalanced outputs. The proposed antenna system was successfully validated, both by simulations and measurements.

## 1. Introduction

Ultra-wide band applications generally include radar, communications, and direction-finding systems. Direction finding usually deals with electromagnetic source localization, including electric sparks in a power distribution system, based on their electromagnetic signature [1].

There are two types of direction-finding methods: amplitude based, and phase-based methods [2]. Specific algorithms can be applied on signals received from antenna arrays, in order to provide accurate direction finding [3].

The simplest amplitude-based method of DF consists in analyzing the received voltage after a mechanical rotation of a directional antenna, considering it as a reference of the source direction. The bearing is then found on a scale placed on the receiving antenna. In that case, the received voltage is displayed as a function of the rotation angle. By using two directional antennas and computing the sum and difference of the received signal amplitudes, one can extract the source bearing without rotating the antenna system [4].

Another amplitude-based technique consists of comparing the signal amplitudes from two orthogonal receiving antennas, in order to find the angle of arrival (AoA) [2]. The method is known as the amplitude comparison technique.

Phase-based approaches include both interferometry and Doppler direction finding [2,4]. Interferometers use antenna arrays in order to find the source direction from the phase differences between the signals received on each antenna. Conversely, a Doppler direction finder compares the phase of the received signal to that of a reference signal with the same central frequency, provided that the radiation pattern is steered either mechanically or electrically, and the frequency modulation occurs on the received signal due to the Doppler effect.

By using antenna arrays, multiple source localization is possible as well [5]. Algorithms for the direction of arrival estimation in the near-field zone with a vector sensor array are proposed in [6]. Moreover, direction and polarization can be extracted, by using improved algorithms for data processing [7].

Several types of radiating systems can be used for direction finding purposes. A direction-finding technique with generic, band limited vector sensors able to discriminate all the six field components is presented in [8]. However, most of the narrow-band designs need only three antennas [9] e.g., one dipole and two orthogonal loops [4]. Three collocated, orthogonal loops can be used instead; wideband operation is made possible by using impedance matching units [10]. A more general analysis covering all possible three-antenna configurations is conducted in [11].

Vector sensor radiating systems including thick wire loops can be used for a fractional bandwidth in the order of 1.5 [12]. Other approaches are based on using dual-band vector sensors in order to provide broadband operation [13]. Moreover, multiband antenna systems can actually be used to localize specific sources, e.g., mobile phones [14]. Planar, spiral shape antennas can provide a more compact solution with a fractional bandwidth figure of around 5 [15,16]. A four-port ultra-wide band (UWB) configuration exhibiting a fractional bandwidth figure of 2 is proposed in [17]. It should be emphasized that multimode antennas have recently been proposed [18], as an alternative to multi-element radiating systems.

As opposed to other direction-finding methods that require large antenna arrays [19], the amplitude comparison technique is based on an antenna system consisting of only two orthogonal loop-type radiators; annular shapes would grant good performance across an ultra-wide band. Since asymmetrical feed lines are generally used in practice, a monopole type antenna might be preferred. However, most of the ring-type monopoles do not preserve the radiation properties of a loop, as they are not actually fed as a loop, but as any other monopole [20,21].

Electric discharges in power distribution systems can generally be modeled as random series of UWB monocycle pulses, with a shape quite similar to the first derivative of the Gauss pulse. Classical narrow band direction finding methods would not lead to an accurate AoA estimation for such electromagnetic sources.

In this paper, we propose a novel UWB antenna system for spark detection and localization in power distribution systems. It consists of two identical, orthogonal padlock-shaped (half-ring) antennas. As opposed to other similar systems [22], each antenna can be asymmetrically driven by using a coaxial cable. This is an advantage over traditional UWB rings which would require an UWB balun. The antenna system was designed to operate with pulses, with a spectrum centered on 250 MHz. The system exhibits very good energy-based figures and an excellent agreement between simulated and measured results can be noted. In order to better quantify the mutual coupling between two UWB antennas with pulsed excitation, we introduced a new descriptor that we called energy-based coupling coefficient. We also show that such a parameter is more relevant to pulsed applications than transmission scattering parameters.

In the last section of our work, we propose a direction-finding methodology using the dual padlock antenna. The methodology combines angle averaging and time gating for a better accuracy.

## 2. Antenna System Design Energy Based Descriptors

The simplest design for amplitude comparison direction finding requires a system of antennas, comprising two vertically oriented loop antennas (ring-shaped), orthogonally arranged, and a “sense” antenna (omnidirectional) used to resolve the “front-back” ambiguity [4].

It has previously been shown [22] that the direction finding of UWB sources can be performed by using the amplitude comparison method, without a sense antenna. In that case, pulse polarities on the orthogonal ring type antenna system show the quadrant of the source direction. Moreover, in the case of spark localization within power plants, the half-space of the incoming wave is usually a priori known.

Thus, the radiating system will only consist of two orthogonal loop elements. Each loop should present a radiation pattern with two lobes over the most of the frequency band, while its nulls are in the antenna plane. For the application under consideration, the polarization of interest is the horizontal one.

The main disadvantage of loop antennas is the balanced input when fed through symmetrical transmission lines. Ultra-wide bandwidth baluns are generally difficult to design and manufacture. In a previous paper [23], we proposed an innovative ultra-wideband, half-ring antenna system sensitive to horizontally polarized electromagnetic field generated by sparks in power plants and energy distribution systems. The novel UWB antenna system has two unbalanced inputs. The excitation of our design was inspired from that used for a folded monopole antenna, i.e., one end of the half ring is connected to the ground plane, and the other one to the inner conductor of the coaxial line. As opposed to folded monopole antennas, our design uses a vertical ground plane since planar antennas are technologically preferred.

**Figure 1:**
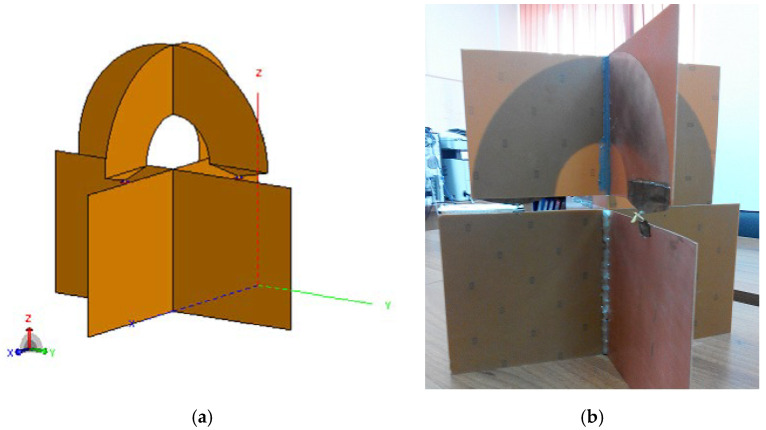
Proposed antenna design: (**a**) Simulation design; (**b**) Experimental antenna. [23].

The system consists of two identical axially crossed “padlock” shaped antennas, as in Figure 1. Thus, the antenna system will retain the advantages of the loop antenna, but can be fed by an asymmetrical coaxial line.

**Figure 2:**
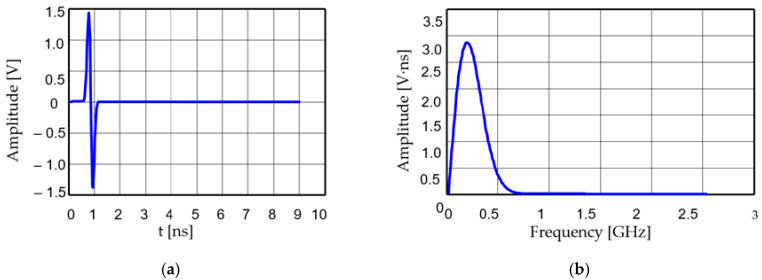
First derivative of a Gaussian function: (**a**) waveform; (**b**) spectrum.

The size of the radiating elements depends on the spectrum of the input pulses generated by the electric discharges. In most cases, the waveform of such a pulse can be assimilated to the first derivative of the Gaussian function presented in Figure 2.

The reflection coefficient in the frequency domain, Γ(ω), is defined as the ratio between the complex amplitudes of the reflected wave and forward wave at the input of the antenna feed line.

The time-domain reflection coefficient is found by applying an inverse Fourier transform, $\gamma \left(t\right)={ℱ}^{-1}\left\{\Gamma \left(\omega \right)\right\}$. The instantaneous reflected voltage at the source can be expressed as a convolution,

(1) $$v_{r}\left(t\right)=\frac{\left(v_{g0}*\gamma \right)\left(t\right)}{2}$$

where $v_{r}\left(t\right)$ is the instantaneous reflected voltage at the source and $v_{g0}\left(t\right)$ is the instantaneous electromotive force of the pulse source. It should be noted that a known, time-domain reflection coefficient, γ(t) gives the reflected output signal for any input signal.

When transmitting or receiving pulsed signals energy-based descriptors should be used instead of classical, frequency-domain antenna parameters [24]. In order to quantify the energy balance at the antenna input, two suitable energy parameters have been defined [25,26]: the pulse reflection coefficient and the pulse matching ratio, respectively. 

The pulse reflection coefficient, g is defined as the square root of the ratio between the energy of the reflected signal and the energy of the forward signal:

(2) $$g=\sqrt{\frac{\mathrm{Reflected}\;\mathrm{signal}\;\mathrm{energy}}{\mathrm{Forward}\;\mathrm{signal}\;\mathrm{energy}}}=\frac{\mathrm{RMS}\left(v_{r}\right)}{\mathrm{RMS}\left(\frac{v_{g0}}{2}\right)}=2\sqrt{\frac{\int_{\mathrm{supp}\;v_{r}\left(t\right)}^{}v_{r}{}^{2}\left(t\right)dt}{\int_{\mathrm{supp}\;v_{g0}\left(t\right)}^{}v_{g0}{}^{2}\left(t\right)dt}=}2\sqrt{\frac{{ℜ}_{v,v_{r}}\left(0\right)}{{ℜ}_{v_{g0},v_{g0}}\left(0\right)}},$$

In (2), $\mathrm{supp}\;v_{r}\left(t\right)$ and $\;\mathrm{supp}\;v_{g0}\left(t\right)$ are the temporal supports of the instantaneous reflected voltage and instantaneous electromagnetic force, respectively; the corresponding autocorrelation functions are denoted by ${ℜ}_{v_{r},v_{r}}\left(0\right)$ and ${ℜ}_{v_{g0},v_{g0}}\left(0\right)$.

As the frequency-domain reflection coefficient, g has a subunitary magnitude; a null would correspond to a perfect matching. It should be highlighted that the pulse reflection coefficient is always defined for a given waveform of the excitation. Moreover, it can be shown that g=|Γ| for sinusoidal signals.

The pulse matching ratio, s [25] is an energy-based descriptor similar to the frequency- domain voltage standing wave ratio (VSWR),

(3) $$s=\frac{1+g}{1-g}$$

The value of this parameter is greater than or equal to 1; a perfect matching is expressed by a s=1. As with other energy-based figures of merit, this parameter is also defined for a given waveform of excitation.

The antenna impulse response is a function of time that characterizes the antenna as a linear system. The input parameter can be the input voltage of a transmitting antenna. The output figure is usually derived from the electric far-field by compensating the propagation effects, i.e., attenuation and delay [4]. The electric far-field can be written as:

(4) $$\left[v_{g}*h_{t}\left(\hat{r}\right)\right]\left(t\right)=rE\left(r,t+r/c_{0}\right),$$

where $v_{g}\left(t\right)$ is the voltage across the antenna input, $h_{t}\left(\hat{r},t\right)$ is the impulse response of the transmitting antenna, which is proportional to its effective height [27], and E(t) is the far, electric field.

The energy gain is an important figure of merit in terms of ultra-wide band radiation, defined as [28]:

(5) $$G\left(\hat{r}\right)=\frac{4\pi \;\mathrm{Energy}\;\mathrm{radiated}\;\mathrm{per}\;\mathrm{unit}\;\mathrm{solid}\;\mathrm{angle}\;\left(\hat{r}\right)}{\mathrm{Total}\;\mathrm{radiated}\;\mathrm{energy}}=\frac{16\pi Z_{0}}{\eta }\frac{{ℜ}_{e_{t\left(\hat{r}\right)},e_{t\left(\hat{r}\right)}}\left(0\right)}{{ℜ}_{v_{g0},v_{g0}}\left(0\right)},$$

where η is the free space wave impedance, and

(6) $$e_{t}\left(\hat{r},t\right)=rE\left(r,t+r/c_{0}\right),$$

where r is the position vector of the field point, $\hat{r}$ is the unit vector of the corresponding direction, and $c_{0}$ is the speed of light.

A new energy-based parameter would be necessary in order to better quantify the mutual coupling for a given activation waveform. An energy-based coupling coefficient can be computed as:

(7) $$c=\sqrt{\frac{\mathrm{Transmitted}\;\left(\mathrm{output}\right)\;\mathrm{energy}}{\mathrm{Forward}\;\left(\mathrm{input}\right)\;\mathrm{energy}}}=\frac{\mathrm{RMS}\left(\nu \right)}{\mathrm{RMS}\left(\frac{\nu_{g}}{2}\right)}=2\sqrt{\frac{\int_{\mathrm{supp}\;\nu \left(t\right)}^{}\nu^{2}\left(t\right)dt}{\int_{\mathrm{supp}\;\nu_{g}\left(t\right)}^{}v_{g}{}^{2}\left(t\right)dt}=}2\sqrt{\frac{{ℜ}_{\nu ,\nu }\left(0\right)}{{ℜ}_{\nu_{g},\nu_{g}}\left(0\right)}},$$

where ν(t) is the output waveform, $\nu_{g}\left(t\right)$ is the input waveform, and ${ℜ}_{\nu_{g},\nu_{g}}$, ${ℜ}_{\nu ,\nu }$ are the autocorrelation functions of the input and output signal, respectively.

## 3. Proposed Direction-Finding Methodology

The shape of the traditional radiation patterns changes from one frequency to another and the nulls do not always occur on the right direction. For antennas with a pulsed excitation, energy-based radiation patterns can be drawn up from the energy gain, defined as in (5).

**Figure 3:**
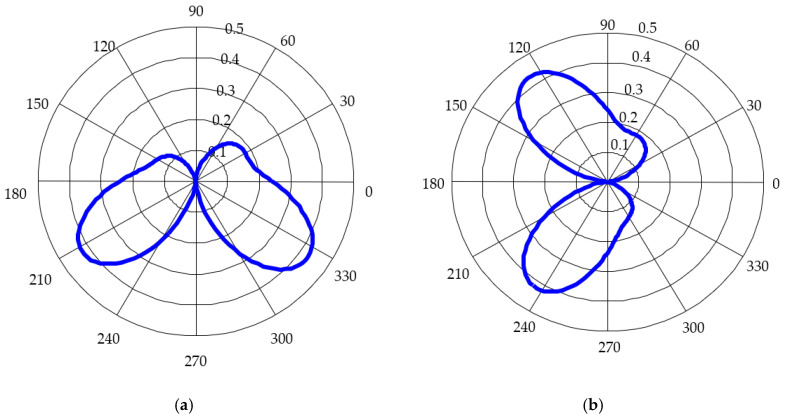
Energy-based pattern diagram: (**a**) First antenna; (**b**) Second antenna.

The antenna system was designed for an excitation proportional to the first derivative of the Gauss function, as in Figure 2. Its spectrum is centered on 250 MHz. The energy radiation patterns for both antennas are shown in Figure 3 when applying the above excitation successively on each antenna.

The nulls of the energy-based radiation patterns occur at φ=90° and 270° for the first antenna, and φ=0° and 180° for the second antenna. An ideal vector sensor consists of two antennas, one with a sine, and the other with a cosine shaped pattern diagram [22]. The angle of arrival can be expressed as the ratio between the signal amplitude at the first antenna output and at the second antenna output, respectively:

(8) $${\mathrm{AoA}}^{\mathrm{ideal}}=\tan^{-1}\frac{A_{1}}{A_{2}}=\tan^{-1}\frac{F_{1}^{ideal}\left(\varphi \right)}{F_{2}^{ideal}\left(\varphi \right)},$$

where $A_{1}$, $A_{2}$ are the received signal amplitudes and $F_{1}^{ideal}$, $F_{2}^{ideal}$ are the ideal radiation characteristics, i.e.,

(9) $$F_{1}^{ideal}\left(\varphi \right)=\left|\sin\varphi \right|,$$

(10) $$F_{2}^{ideal}\left(\varphi \right)=\left|\cos\varphi \right|.$$

As shown in Figure 3, the real energy-based radiation patterns are not proportional to the sine and cosine of the angle of incidence, respectively. By defining

(11) $$R\left(\varphi \right)=\frac{A_{1}}{A_{2}}=\frac{F_{1}^{real}\left(\varphi \right)}{F_{2}^{real}\left(\varphi \right)},$$

The AoA is then found,

(12) $${\mathrm{AoA}}^{\mathrm{real}}≅R^{-1}\left(\frac{A_{1}}{A_{2}}\right).$$

**Figure 4:**
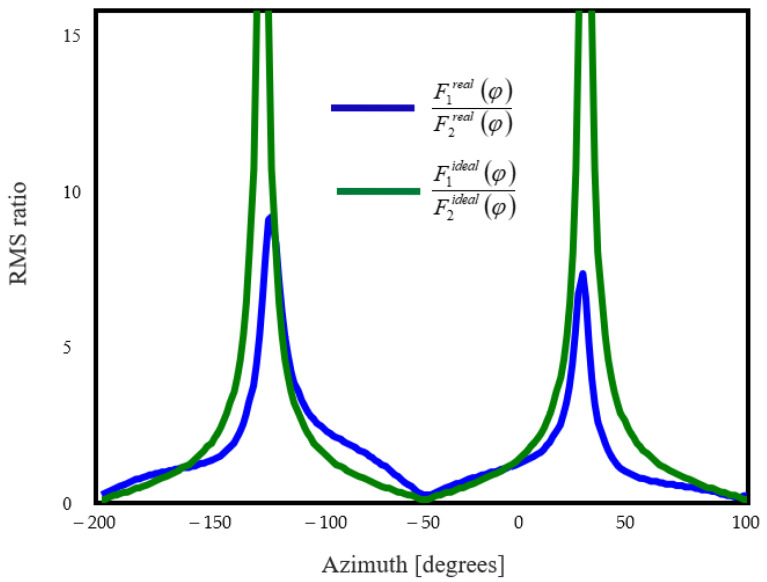
Ratio between RMS of the received signals.

Figure 4 presents a comparison between the real and ideal radiation characteristics.

As Figure 4 shows, computing the AoA by simply evaluating the arctangent of the signal ratio would lead to errors. Since R(φ) may not result in an analytical form, its inverse can be computed by using a lookup table.

## 4. Results: Antenna System

**Figure 5:**
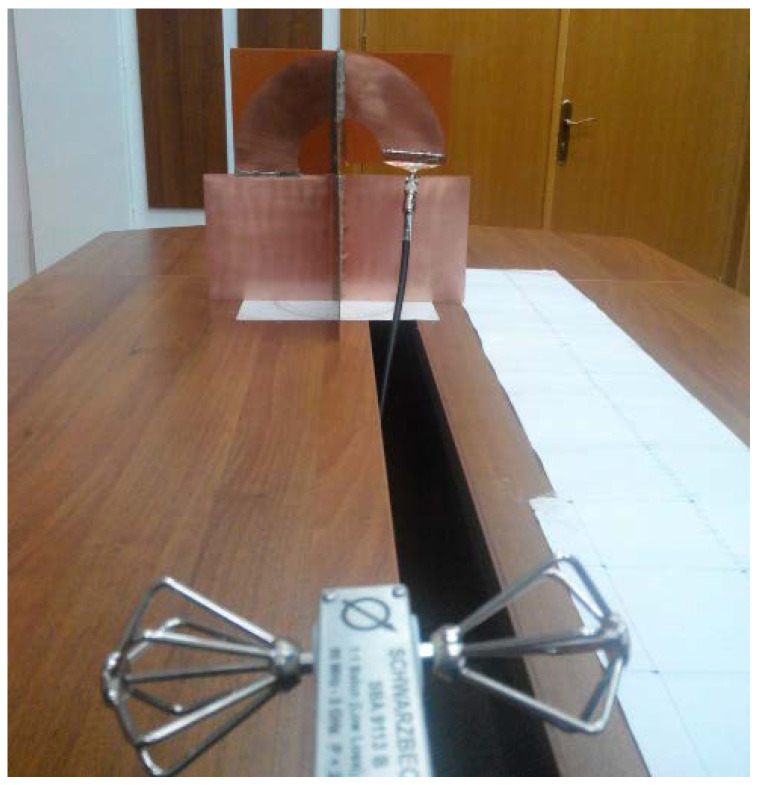
Measuring setup [23].

The measuring setup (Figure 5) for the frequency-domain gain consists of the antenna system under test, a calibrated antenna, and a vector network analyzer.

**Figure 6:**
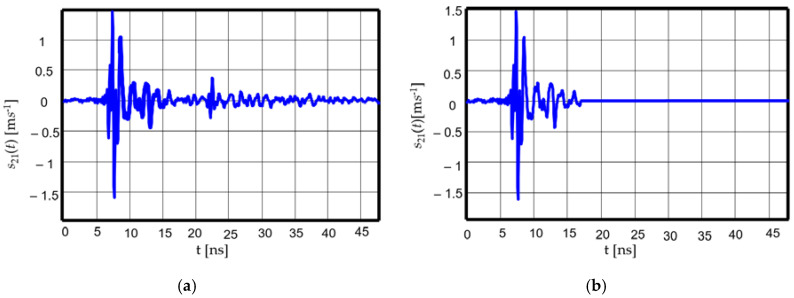
Time-domain response: (**a**) before windowing; (**b**) after windowing [23].

Since we measured an ultra-wide band radiating system, an anechoic chamber was not necessary. An inverse Fourier transform was performed on the measured data, and the result was windowed in the time-domain, as the system response was short enough compared to the shortest indirect propagation path. Figure 6 gives a sample of time-domain response before (a) and after (b) windowing. In Figure 6a, one can easily discriminate the system response from the effect of the multipath propagation.

In order to find the antenna gain from the measured S parameters, we evaluate the ratio between the received and transmitted power with the Friis formula:

(13) $$\frac{P_{r}}{P_{t}}=G_{t}G_{r}\left(\frac{\lambda }{4\pi d}\right),$$

where d is the distance between the two antennas, $G_{t}$ is the gain of the calibrated (transmitting) antenna and $G_{r}$ is the gain of the measured (receiving) antenna.

**Figure 7:**
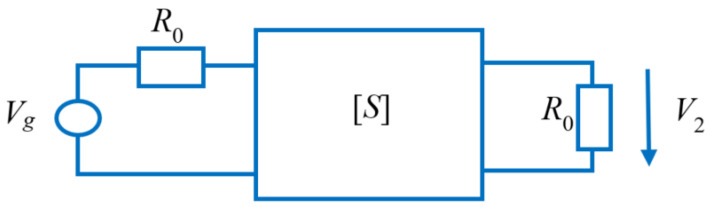
Equivalent two-port circuit.

By assimilating the entire setup to a two-port device terminated on $R_{0}=50\;\Omega$, as shown in Figure 7, the transfer function can be written as:

(14) $$S_{21}=\frac{2V_{2}}{V_{g}}$$

By using the Friis formula and taking into account the impedance mismatch at each antenna [29]

(15) $$\sqrt{G_{t}G_{r}}=\frac{4\pi d\cdot \left|S_{21}\right|}{\lambda \;\left|1-S_{22}\right|}\cdot \sqrt{\frac{R_{0}}{R_{a2}\left(f\right)\cdot \left(1-{\left|S_{11}\right|}^{2}\right)}}$$

where $R_{a2}\left(f\right)$ is the radiation resistance of the antenna under test.

Consequently, the gain of the antenna under test is

(16) $$G_{r}=\frac{1}{G_{t}}{\left(\frac{4\pi d}{\lambda }\right)}^{2}\frac{R_{0}}{R_{a2}\left(f\right)}\frac{{\left|S_{21}\right|}^{2}}{{\left|1-S_{22}\right|}^{2}\left(1-{\left|S_{11}\right|}^{2}\right)}$$

where $S_{21}$ is the Fourier transform of the impulse response, after windowing the signal.

**Figure 8:**
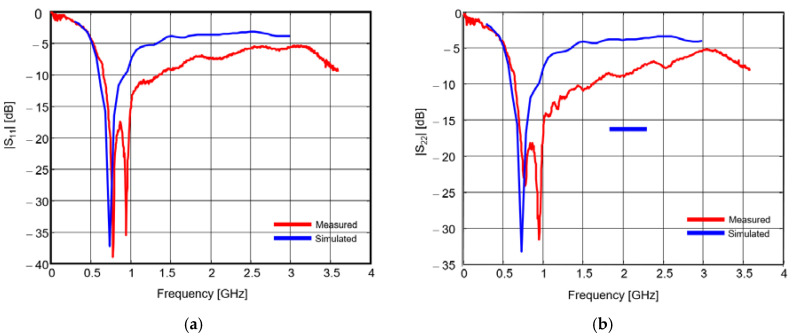
Reflection coefficients: (**a**) First antenna; (**b**) Second antenna [23].

**Figure 9:**
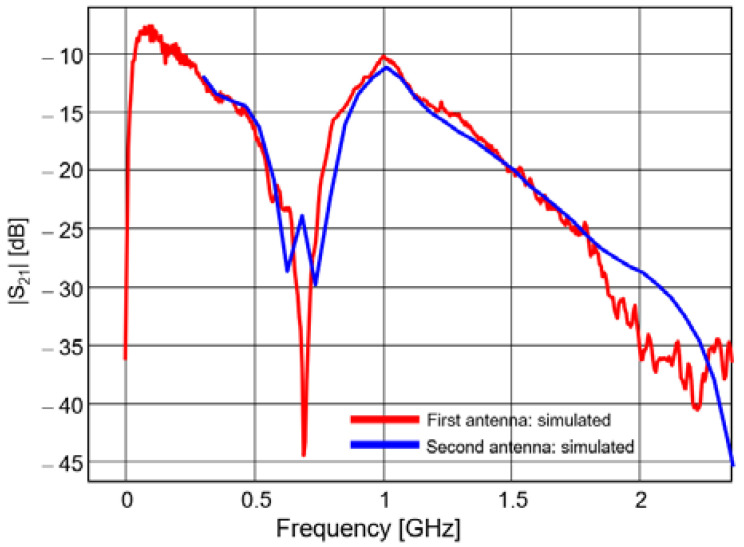
Transfer function between the two padlock antennas [23].

The S parameters of the proposed antenna system are presented in Figure 8 and Figure 9. The simulated results (in blue) are compared to measured results (in red).

A very good agreement can be noted between the measured and simulated transmission parameters; conversely, there are some discrepancies between the simulated and measured reflection parameters. Those discrepancies are mainly due to the lack of accuracy of our simulator for modeling the excitation. The measured reflection coefficient is actually better than that resulting from simulation, as its magnitude is below −10 dB between 700 MHz and 1.4 GHz, and below −5 dB between 500 MHz and 2.2 GHz.

The magnitude of the transmission coefficient reaches a global maximum of −8 dB around 100 MHz and a local maximum of −10 dB around 1 GHz. That figure might not be satisfying for narrow band applications at such frequencies; the mutual coupling is quite high and that would impinge on the orthogonality. However, for pulsed waveforms, the mutual coupling should be assessed in straight correlation with the spectral power density of the signal.

Energy-based descriptors are actually more relevant for pulsed applications than frequency- domain parameters. For our antenna system, and for the first derivative of a Gauss function (Figure 2), as an excitation with a spectrum centered on 250 MHz, we found *g* = −5.51 dB and *s* = 3.2567, respectively. The energy-based coupling coefficient is −17.86 dB.

**Figure 10:**
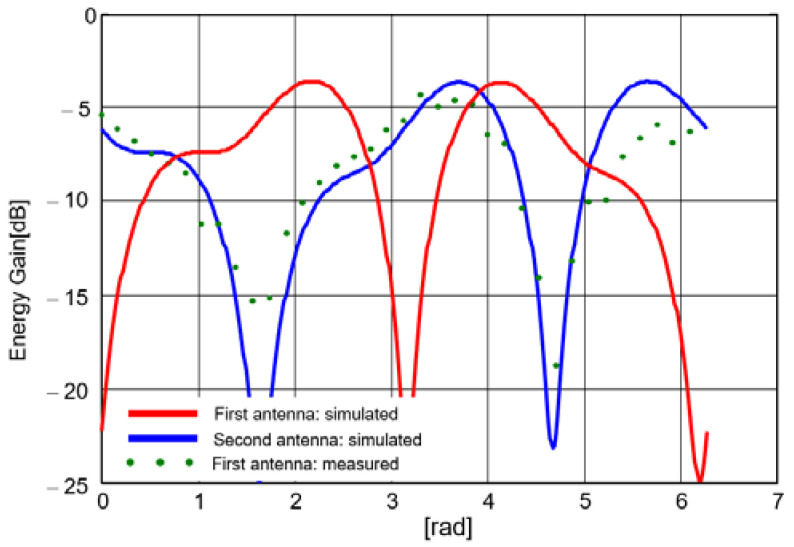
Energy gain pattern diagram [23].

In order to measure the energy-based gain, the antenna was rotated horizontally with an angular pitch of 10°. Figure 10 presents the energy-gain pattern diagrams for each padlock antenna versus simulated gain. The diagrams were plotted for θ=90° and horizontal polarization.

A very good agreement between simulations and measurements can be noted. There are deep extinctions of the pattern diagrams in the plane of each antenna, which makes the proposed system appropriated to UWB direction finding purposes.

## 5. Results: Direction Finding Approach

**Figure 11:**
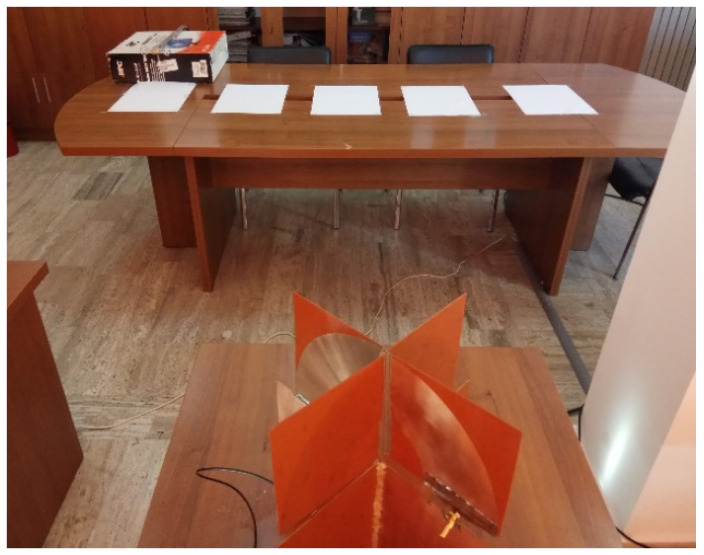
Direction finding approach: measurement setup.

**Figure 12:**
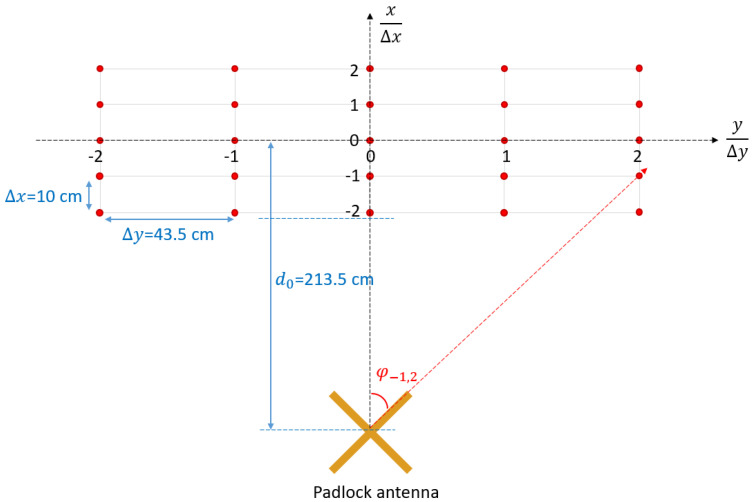
Matrix of measuring positions.

In order to validate our UWB direction finding approach, we utilized a setup consisting of the padlock antenna system, a calibrated biconical antenna and a vector network analyzer (Figure 11). We measured two transfer functions, i.e., between the probe antenna and each padlock radiator, with the calibrated antenna placed successively in a matrix of 25 positions, as in Figure 12.

The measurements were performed in a multipath environment. In practice, such a matrix of measuring points would correspond to an average based methodology of direction finding with the aim to reduce the effect of the multipath propagation [30]. That is, one can take the middle row in Figure 12, as successive positions of the electromagnetic source to be found. The columns would actually be successive positions of the padlock antenna, and the measured azimuth angles in one column are subject to averaging, in order to accurately estimate the AoA. Additionally, a time domain gating is performed on each received waveform, in order to further improve the accuracy.

It should be noted that, when measuring the transfer function of one radiator, the other one was terminated on a 50 ohm load.

**Figure 13:**
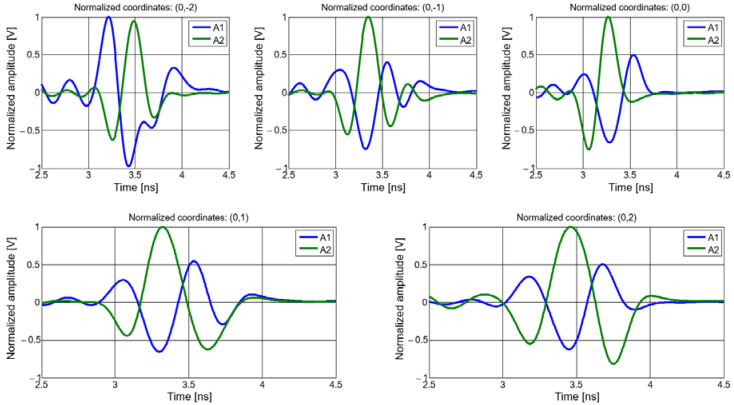
Waveforms of the received signals: middle (reference) row.

In Figure 13, we show the waveforms of the signals received on the two padlock radiators for the middle (reference) row in the matrix of measuring positions.

**Figure 14:**
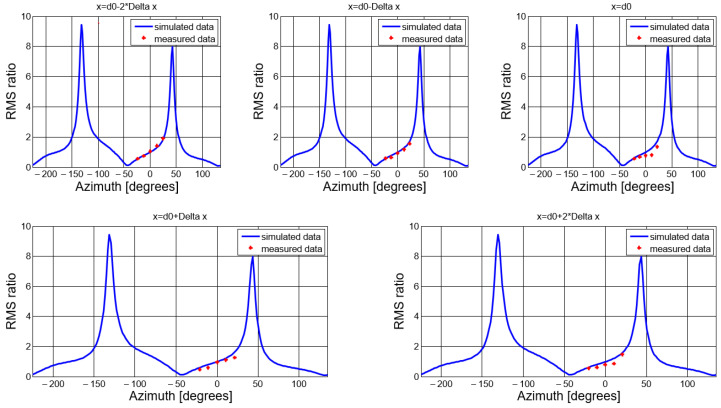
Ratio between RMS voltages of the received signals as a function of azimuth angle: measured and simulated results.

We computed the ratio between the RMS of the two received waveforms for each measuring position, and compared them to the simulated results in Figure 4. The comparison is shown in Figure 14.

Real and estimated azimuth angles are given in Table 1 and Table 2, respectively.

A mean error figure can be evaluated for each direction as

(17) $$\bar{\varepsilon_{j}}=\frac{1}{2N+1}\overset{N}{\underset{i=-N}{\sum }}\left({\hat{\varphi }}_{ij}-\varphi_{0j}\right)$$

The error vector is shown in Table 3. The resulting overall mean error is 5.32°.

## 6. Conclusions

We introduced a new dual padlock antenna designed for UWB applications, namely for spark localization within power plants and distribution systems. The antenna system was conceived for an amplitude comparison method. The main advantage over other loop-type antennas for direction finding consists of an unbalanced input that makes it possible to use asymmetrical feed lines without inserting a balun. The antenna system was simulated and then manufactured and measured. The characterization was performed by using both frequency-domain parameters and energy-based descriptors, such as pulse reflection coefficient, pulse matching ratio, energy gain and the energy-based coupling coefficient that we have presented in a separate section.

A good agreement between measured and simulated data could be noted. For the first derivative of a Gauss function, as an excitation with a spectrum centered on 250 MHz, we found a pulse matching ratio of 3.2567, and an energy-based coupling coefficient of −17.86 dB. The energy pattern diagrams for θ = 90° and horizontal polarization have deep nulls in the planes containing each antenna, which makes our system appropriated to direction finding.

The proposed direction-finding approach was validated by locating a calibrated antenna successively placed in a matrix of 25 positions.

We found an overall, mean absolute error of 5.32°. The discrepancies between simulated and measured azimuth error are mainly due to the finite bandwidth of the measured transfer functions that results in ripples before and after the time support of the received signals that contribute to the RMS.

We should point out that monitoring electric sparks is based on random signals. Consequently, moving the padlock antenna away from the electromagnetic source may result in received signals with a spectral power density below the noise level for some measuring positions. Detection and locating may be improved by using a collaborative system [31,32], with several padlock antennas rather than one antenna scanning.

## Figures and Tables

**Table 1:** Real azimuth angles.

$\varphi_{ij}$ [°]	j=−2	j=−1	j=0	j=1	j=2
i=−2	24.21	12.67	0.00	−12.67	−24.21
i=−1	23.15	12.07	0.00	−12.07	−23.15
i=0	22.17	11.52	0.00	−11.52	−22.17
i=1	21.27	11.01	0.00	−11.01	−21.27
i=2	20.43	10.55	0.00	−10.55	−20.43

**Table 2:** Estimated azimuth angles.

${\hat{\varphi }}_{ij}$ [°]	j=−2	j=−1	j=0	j=1	j=2
i=−2	25.20	16.20	1.80	−14.40	−23.40
i=−1	19.80	9.00	−3.60	−19.80	−21.60
i=0	14.40	−10.80	−10.80	−16.20	−23.40
i=1	12.60	5.40	−1.80	−21.60	−27.00
i=2	18.00	−7.20	−10.80	−19.80	−23.40

**Table 3:** Azimuth mean error vector.

j	−2	−1	0	1	2
$\bar{\varepsilon_{j}}$ [°]	4.17	9.00	5.04	6.84	1.59

## References

[1] Kozlowski A., Barski M., Stuchly S. (1990). Measurement of the Radiated Electric Fields from Electrostatic Discharge.

[2] Brinegar C. (2000). Passive Direction Finding: Combining Amplitude and Phase Based Methods.

[3] Thiele G.A. (1975). Electromagnetic Direction Finding Techniques.

[4] (2016). Radiomonitoring & Radiolocation Catalog. https://cdn.rohde-schwarz.com/ru/downloads_45/common_library_45/brochures_and_datasheets_45/Radiomonitoring_and_Radiolocation_Catalog.pdf.

[5] Schmidt R.O. (1986). Multiple emitter location and signal parameter estimation. IEEE Trans. Antennas Propag..

[6] Ma H., Tao H., Kang H. (2016). Mixed far-field and near-field source localization using a linear electromagnetic-vector-sensor array with gain/phase uncertainties. IEEE Access.

[7] Li J. (1993). Direction and polarization estimation using arrays with small loops and short dipoles. IEEE Trans. Antennas Propag..

[8] Nehorai A., Paldi E. (1994). Vector-sensor array processing for electromagnetic source localization. IEEE Trans. Signal Process..

[9] Wong K.T. (2001). Direction finding/polarization estimation–dipole and/or loop triad(s). IEEE Trans. Aerosp. Electron. Syst..

[10] Musicant A., Almog B., Oxenfeld N., Shavit R. (2015). Vector sensor antenna design for VHF band. IEEE Antennas Wirel. Propag. Lett..

[11] Yuan X., Wong K.T., Xu Z., Agrawal K. (2012). Various compositions to form a triad of collocated dipoles/loops, for direction finding and polarization estimation. IEEE Sens. J..

[12] Appadwedula S., Keller C.M. (2006). Direction-Finding Results for a Vector Sensor Antenna on a Small UAV.

[13] Lominé J., Morlaas C., Imbert C., Aubert H. (2015). Dual-band vector sensor for direction of arrival estimation of incoming electromagnetic waves. IEEE Trans. Antennas Propag..

[14] Sarkis R., Craeye C., Férréol A., Morgand P. (2009). Design of Triple Band Antenna Array for GSM/DCS/UMTS Handset Localization.

[15] Bellion A., Meins C.L., Vergonjanne A.J., Monediere T. A New Compact Dually Polarized Direction-Finding Antenna on the UHF Band. Proceedings of the 2008 IEEE Antennas and Propagation Society International Symposium.

[16] Pack R.N., Lasser G., Filipovic D.S. (2018). Performance characterization of four-arm MAW spiral antennas for digital direction-of-arrival sensing. IEEE Trans. Antennas Propag..

[17] Duplouy J., Morlaas C., Aubert H., Potier P., Pouliguen P., Djoma C. (2019). Wideband and reconfigurable vector antenna using radiation pattern diversity for 3-D direction-of-arrival estimation. IEEE Trans. Antennas Propag..

[18] Pohlmann R., Zhang S., Jost T., Dammann A. Power-based direction-of-arrival estimation using a single multi-mode antenna. Proceedings of the 14th Workshop on Positioning, Navigation and Communications (WPNC).

[19] Marrocco G., Galletta G. (2010). Hermite-rodriguez UWB circular arrays. IEEE Trans. Antennas Propag..

[20] Deodhar K., Baxi P., Naik A., Gupta R. (2007). Printed Annular Ring Monopole Antenna for UWB Applications.

[21] Guo L., Rehman M., Liang J., Chen X., Parini C. (2007). A Study of Cross Ring Antenna for UWB Applications.

[22] Schantz H.G. Smart antennas for spatial RAKE UWB systems. Proceedings of the IEEE Antennas and Propagation Society International Symposium.

[23] Topor R., Tamas R.D. A Novel Dual-Padlock UWB Antenna System. Proceedings of the 2015 iWAT.

[24] Allen O.E., Hill D.A., Ondrejka A.R. (1993). Time-domain antenna characterizations. IEEE Trans. Electromagn. Compat..

[25] Tamas R., Babour L., Fond E., Alexa M., Slamnoiul G., Cosereanu L., Saguet P., Chilo J. Energy-based input reflection coefficient for the characterization of ultra-wide band antennas. Proceedings of the 2008 International Workshop on Antenna Technology: Small Antennas and Novel Metamaterials.

[26] Topor R.E., Bucuci S., Tamas R.D., Danisor A., Dumitrascu A., Berescu S. (2014). Direction Finding Antenna System for Spark Detection and Localization.

[27] Shlivinski A., Heyman E., Kastner R. (1997). Antenna characterization in the time domain. IEEE Trans. Antennas Propag..

[28] Tamas R., Babour L., Fond E., Slamnoiu G., Chilo J., Saguet P. (2008). Cylindrical dipoles as ultra-wide band antennas: An energy-based analysis. Microw. Opt. Technol. Lett..

[29] Anchidin L., Ilie C.-A., Bucuci S., Tamas R., Caruntu G. (2018). A New Insight on the Distance Averaging Method: Linear Scanning Versus Matrix Scanning.

[30] Tamas R.D., Deacu D., Vasile G., Ioana C. (2014). A method for antenna gain measurements in nonanechoic sites. Microw. Opt. Technol. Lett..

[31] Yan J., Pu W., Zhou S., Liu H., Bao Z. (2020). Collaborative detection and power allocation framework for target tracking in multiple radar system. Inf. Fusion.

[32] Siriwongpairat W.P., Han Z., Liu K.J.R. (2007). Power controlled channel allocation for multiuser multiband UWB systems. IEEE Trans. Wirel. Comm..

